# Structural Evolution of Low-Molecular-Weight Poly(ethylene oxide)-*block*-polystyrene Diblock Copolymer Thin Film

**DOI:** 10.1155/2013/539457

**Published:** 2013-10-31

**Authors:** Hui Wu, Xiaohua Huang

**Affiliations:** ^1^College of Material Engineering, Fujian Agriculture and Forestry University, Fuzhou 350002, China; ^2^Key Laboratory of New Processing Technology for Nonferrous Metal & Materials of Ministry of Education, Guangxi Scientific Experiment Center of Mining, Metallurgy and Environment, and School of Material Science and Engineering, Guilin University of Technology, Guilin 541004, China

## Abstract

The structural evolution of low-molecular-weight poly(ethylene oxide)-*block*-polystyrene (PEO-*b*-PS) diblock copolymer thin film with various initial film thicknesses on silicon substrate under thermal annealing was investigated by atomic force microscopy, optical microscopy, and contact angle measurement. At film thickness below half of the interlamellar spacing of the diblock copolymer (6.2 nm), the entire silicon is covered by a polymer brush with PEO blocks anchored on the Si substrate due to the substrate-induced effect. When the film is thicker than 6.2 nm, a dense polymer brush which is equal to half of an interlamellar layer was formed on the silicon, while the excess material dewet this layer to form droplets. The droplet surface was rich with PS block and the PEO block crystallized inside the bigger droplet to form spherulite.

## 1. Introduction

The formation of diverse patterns in polymer thin films is of considerable technological and scientific importance in coatings, adhesives, photoresists, electronics, biomaterials, and optical devices [1–5]. By thermal [2, 6] or solvent [7, 8] annealing, melt-wetting templates [9, 10], and nanoimprint lithography [11, 12], patterned surface with unique topography and properties can be constructed. For example, poly(**ε**-caprolactone) (PCL) film with hierarchical nanowire patterns fabricated by melt-wetting anodic aluminum oxide templates showed a higher capability of protein adsorption and better cell growth than that with smooth surface [9].

The chemical linkage of two distinct blocks in diblock copolymers results in new material properties which are different from the individual homopolymers [6]. When the blocks spontaneously microphase-separate, a wide range of structures, such as lamellae, double gyroids, cylinders, and spheres, is formed in block copolymers depending on copolymer composition and preparation conditions [6, 13–15]. For the diblock copolymer in thin films, many researches have been exclusively devoted toward understanding and controlling microstructural and topographical features [16–30]. Due to a combination of block/interfacial interactions and entropy, the morphologies and properties of thin films with different thicknesses differ appreciably from bulk materials. The ultrathin films of a polystyrene-*block*-poly(2-vinylpyridine) (PS-*b*-P2VP) diblock copolymer on mica formed a chemically heterogeneous surface pattern [16]. Thin diblock copolymer films under thermal annealing exhibited a hierarchy of morphologies because of the interfacial interactions between the copolymers and substrate and the complex coupling and competition between dewetting and microphase separation [2, 17–20]. Above the bulk order-disorder transition temperature (*T*_ODT_) of symmetric polystyrene-*block*-poly(methyl methacrylate) (PS-*b*-PMMA), the mismatching of film thickness and bulk periodicity result in the hierarchical formation, ranging from spinodal morphology to islands/holes [2, 17]. To achieve the minimization tendency of surface tension, superimposed lamellae are formed in poly(styrene)-*block*-poly(**ε**-caprolactone) (PS-*b*-PCL) thin films when annealed lower than *T*_ODT_ [18].

Poly(ethylene oxide) (PEO) is an effective material for the prevention of protein adsorption and platelet adhesion [31, 32]. Therefore, PEO-based block copolymer thin films have been proposed to be an important biomaterial in bioengineering for antifouling applications [1]. The combination of patterning ability and biological compatibility has made these kinds of block copolymers interesting and promising materials for the design of biointerfaces with novel properties. To make a fundamental understanding of the interfacial properties and morphology of the PEO-based copolymer thin film, in this research, the structural evolution of a low-molecular-weight (5000–4300) poly(ethylene oxide)-*block*-polystyrene (PEO-*b*-PS) diblock copolymer thin films on silicon substrate was demonstrated. The *T*_ODT_ of this copolymer is lower than the glass transition temperature of amorphous PS block and the melting temperature of semicrystalline PEO block. The composition and topography of patterned surface with various initial film thicknesses under thermal annealing were investigated by atomic force microscopy, optical microscopy, and contact angle measurement.

## 2. Experimental Section

### 2.1. Sample Preparation

The PEO-*b*-PS diblock copolymer was synthesized via atom transfer radical polymerization (ATRP) using bromotelechelic PEO macroinitiator. The PEO block had a number-average molecular weight (*M*_*n*_) of 5000 g/mol and a polydispersity of 1.02 as characterized by gel permeation chromatography (GPC) using PS standards. The  *M*_*n*_  of PS block was 4300 g/mol as determined by proton nuclear magnetic resonance (^1^H NMR). The polydispersity of final diblock copolymer was 1.12 using GPC with a universal calibration.

To prepare the PEO-*b*-PS thin film with different thickness, the PEO-*b*-PS diblock copolymer powders were dissolved into purified chloroform (CHCl_3_) in concentrations ranging from 0.01 to 0.5 wt%. The solutions were stored over 24 h to obtain sufficient dissolution. The thin film samples were prepared by spin-casting the solution on freshly cleaned silicon wafers at a speed of 3500 rpm for 40 s and dried under vacuum for 2 h at room temperature to remove the solvent residuals. To study the structural evolution of PEO-*b*-PS diblock films under thermal annealing, the films were annealed for 8 h at 393 K under vacuum to achieve a stable thermal equilibrium state and then slowly cooled to room temperature (cooling rate Ca. 20 K/h).

### 2.2. Spectroscopic Ellipsometry

The thickness of freshly prepared films on a silicon wafer was measured using a spectroscopic ellipsometer (Horiba JOBIN YVON Uvisel). The ellipsometric data were collected with a spectral range from 300 to 800 nm at incidence angle of 70°. The film thickness is the average results of three measurements at different positions in the film.

### 2.3. Polarized Light Microscopy

The polarized light microscopy (PLM) observations of the thin films were carried out using a Leica DMLP microscope equipped with a Linkam THMSE-600 hot stage. To observe the structure evolution of PEO-*b*-PS film under thermal annealing, the samples were rapidly heated to 393 K at a rate of 120 K/min and annealed for 8 h.

### 2.4. Atomic Force Microscopy

The surface morphology of all the thin films was characterized by an atomic force microscope (SPA300HV/SPI3800N Probe Station, Seiko Instruments Inc., Japan) in tapping mode. The sample was mounted on a sample stage driven by a piezo tube scanner, which was calibrated with standard gratings before use. A silicon microcantilever (spring constant 42 N/m and resonance frequency ~285 KHz, Olympus Co., Japan) was used for scan. The scan rate was 1.0 Hz and scan line pixels were 256.

### 2.5. Surface Analysis

Water contact angles were measured using a Kruss DSA10-MK2 drop shape analyzer at room temperature using Milli-Q water as the probe fluid (5 *μ*L). Each contact angle value reported was an average of at least five independent measurements.

## 3. Results

The initial thickness of PEO-*b*-PS film is controlled by varying the diblock copolymer solution concentration. The films with thickness of 2.3, 5.8, 10.7, 25.6, and 43.9 nm, as determined prior to thermal annealing by ellipsometry, were obtained by spin-casting CHCl_3_ PEO-*b*-PS solution with concentration of 0.02, 0.05, 0.1, 0.3, and 0.5 wt.%, respectively. The as-prepared spin-cast films are flat and featureless, indicating that the block copolymers are in a disordered state.

**Figure 1:**
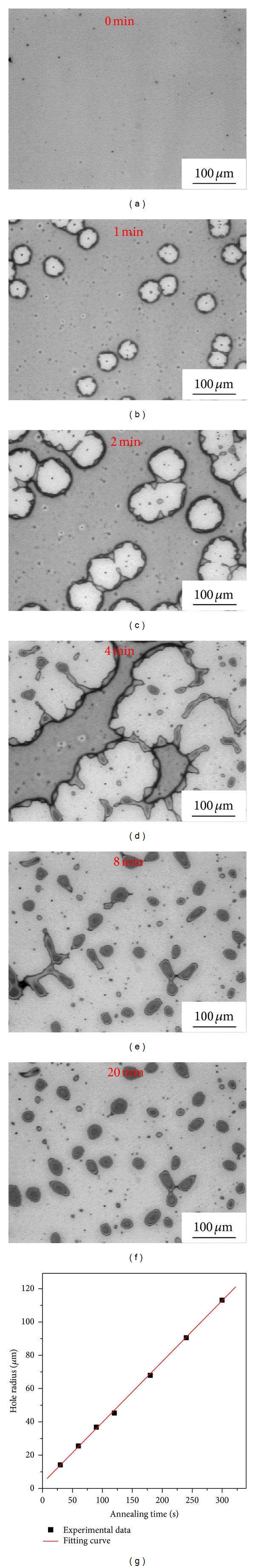
Optical micrographs of a PEO-*b*-PS film with initial thickness of 43.9 nm annealed at 393 K for (a) 0 min, (b) 1 min, (c) 2 min, (d) 4 min, (e) 8 min, and (f) 20 min. And (g) radius of hole as a function of annealing time.

To show the surface structural evolution of PEO-*b*-PS film under thermal annealing, the PLM images of a diblock copolymer film with initial thickness of 43.9 nm annealed at 393 K were recorded continuously (Figures 1(a)–1(f)). As the heating proceeds, the smooth film (Figure 1(a)) surface ruptured randomly and created randomly distributed holes (Figure 1(b)). The rims can be seen as dark rings around the holes. The holes have grown gradually and pushed the rim of liquid copolymer ahead (Figure 1(c)). When two moving rims came into contact, they merged forming cellular structures (Figure 1(d)). The resulting liquid ribbons were unstable and eventually break into randomly dispersed discrete droplets (Figure 1(e)) spontaneously throughout the surface of the film due to Rayleigh instabilities.

Figure 1(g) showed the rate of the holes growth at 393 K measured from the PLM images which can be fitted with linear function, indicating that hole growth experienced the viscous flow regime [18]. The growth rate was 0.37 *μ*m/s, which is much higher than that of PS-*b*-PCL block copolymer (ca. 0.015 *μ*m/s) [18]. This indicates a higher mobility of PS-*b*-PEO melt because of the lower molecular weight.

**Figure 2:**
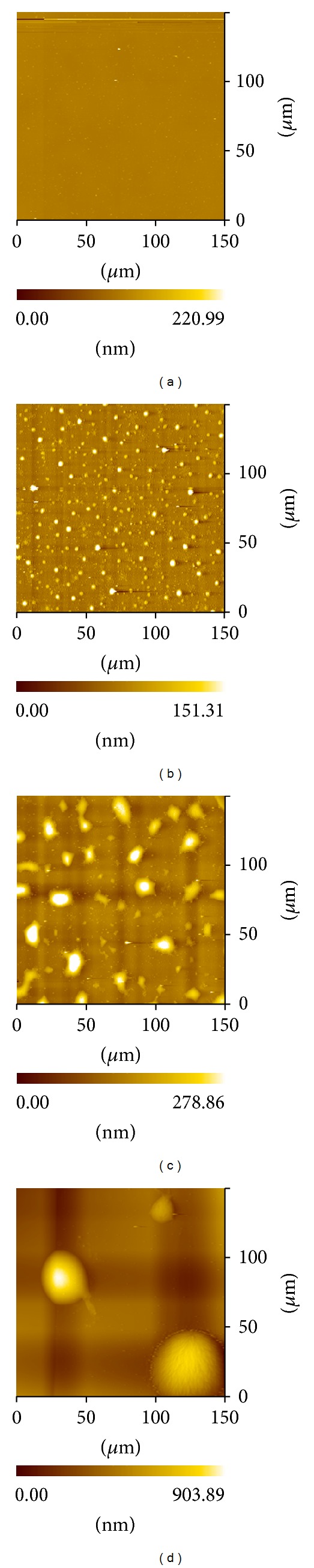
AFM topographies of PEO-*b*-PS film after annealing for 8 h at 393 K with initial thickness of (a) 5.8 nm, (b) 10.7 nm, (c) 25.6 nm, (d) 43.9 nm.

After it was fully annealed to the equilibrium state at 393 K and slowly cooled to room temperature, the AFM topographies of PEO-*b*-PS films with different initial film thickness with scan area of 150 *μ*m × 150 *μ*m were shown in Figure 2. In Figure 2(a), no obvious droplets were observed in the film of initial thickness of 5.8 nm. When the initial film thickness increased to 10.7 nm (Figure 2(b)), discrete droplets of dewetted film are formed. For the initial film thickness of 10.7, 25.6, and 43.9 nm, the average droplet diameter measured by AFM is about 1.8, 4.0, and 40.8 *μ*m, respectively. The average size of the droplet increased with increasing the thickness of the copolymer film (Figures 2(b)–2(d)).

**Figure 3:**
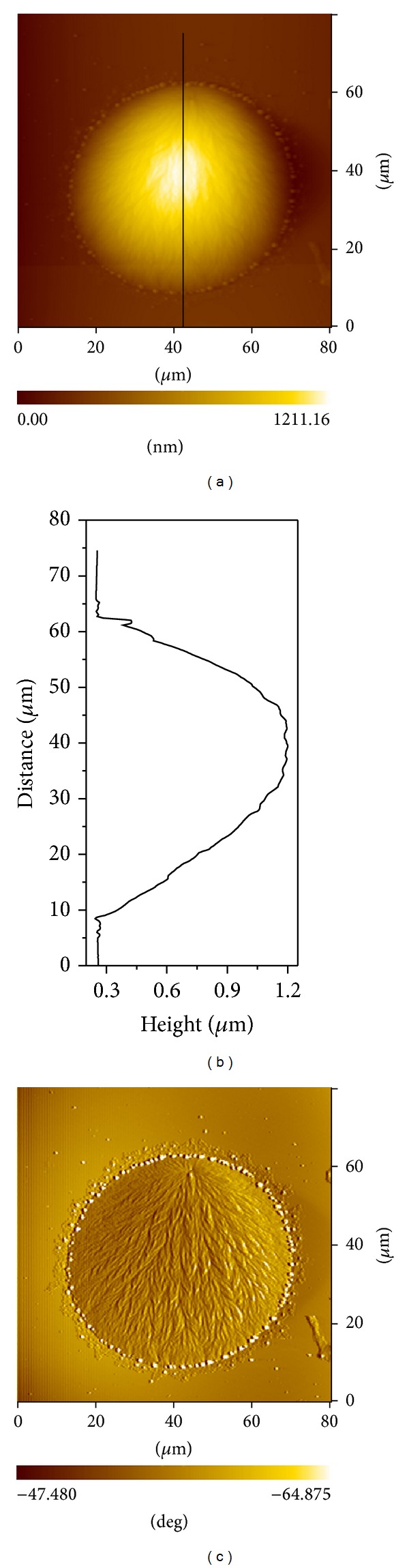
AFM topography (a) and phase image (c) of droplet in the film of initial thickness of 43.9 nm (b) is the corresponding cross-section line profiles in (a).

**Figure 4:**
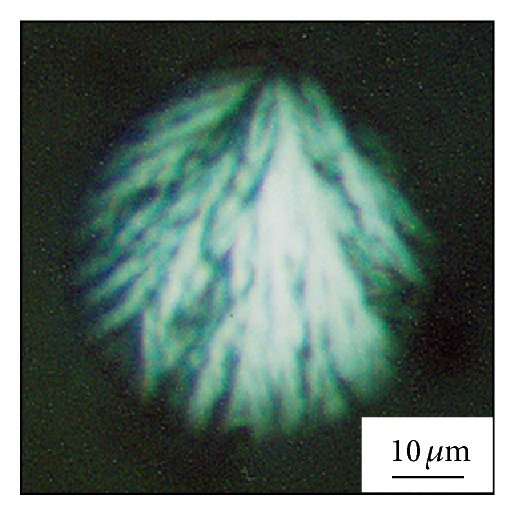
PLM image of spherulite in the droplet in the film of initial thickness of 43.9 nm.

In Figure 2(d), the smaller droplet in the left is in the amorphous state, while the bigger droplet in the bottom right showed a typical single spherulitic structure. The bigger droplet in Figures 3(a) and 3(c) is the magnification of the crystallized droplets in Figure 2(d). Tiny droplets can be observed around the big droplet in the phase image (Figure 3(c)). On the top of big the droplet, a nucleus can be clearly observed, and it develops into a spherulite with a bunch of branches filling the whole droplet volume. Under crossed polarizers, the PLM image of droplet in Figure 4 appears bright, which confirms the formation of spherulite in the droplet. This observation is similar to the spherulites formed within separate PEO droplets which dewetted on the PS thin film [33]. However, the spherulitic structure formed in the droplets of low-molecular-weight PEO-*b*-PS is different from the structure of concentric circular superimposed or terraced lamellar layers which stack on top of each other [21, 34].

**Figure 5:**
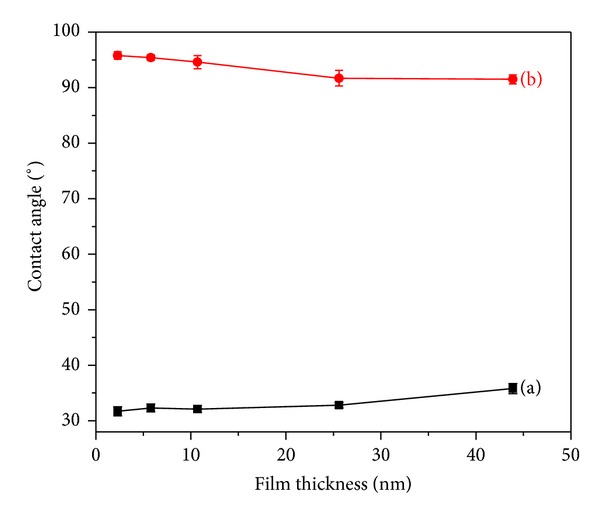
The contact angles of PEO-*b*-PS films with different initial thickness (a) before and (b) after annealing at 393 K for 8 h.

In order to identify the surface composition in the as-prepared films and annealed films, water contact angle measurement was carried out.  Figure 5 mapped a plot of the changing contact angle value before and after annealing in various thickness films. The water contact angle of the silicon substrate with a native SiOx surface layer, the spin-coated homopolymer films of PS, and PEO are measured to be 21.6°, 95.8°, and 21.0°, respectively. For the PEO-*b*-PS film with different thickness before annealing, the average contact angle value is about Ca. 34°. This value is close to that of homopolymer PEO film, showing that the surface of as-prepared film is hydrophilic and is rich with PEO block. After it was annealed at 393 K for 8 h, the contact angle value increased to ~94°, which is close to that of homopolymer PS film, indicating that the surface changed to hydrophobic and the surface is rich with PS block.

## 4. Discussion

The nature and strength of the interfacial interactions at the asymmetric interfaces (block/solid and block/air interface) significantly influence the block copolymer thin film morphology, phase separation, and orientation of microstructures. If one block in the symmetric diblock copolymers is preferred for any of the interfaces (substrate or free interface), a lamellae orientation parallel to the substrate can be observed [22]. For the films of initial thickness of 2.3 and 5.8 nm after annealing, the film surface was kept smooth and no droplets were seen during the AFM measurement. This observation suggests that dewetting is prohibited and a copolymer layer is adsorbed on the surface of oxidized silicon.

Because PEO is adsorbed on the hydroxyl groups on the surface of the silicon oxide substrate, the PEO blocks have preferential wettability and stronger affinity to the Si wafer. And the surface energy of PS block (*γ*_PS_ = 40.7 mN/m) is lower than that of PEO block (*γ*_PEO_ = 42.9 mN/m). This helps the PS component with the lower surface energy to aggregate to the free air/film surface. The overall results are that these asymmetric boundary conditions cause the PEO block to preferentially segregate to the substrate while the PS block to the air interface. The copolymer chains oriented along the normal of the film/substrate interface, forming a copolymer brush with a lamellar structure parallel to the interface.

However, the droplets formed in the film of initial thickness higher than 10.7 nm. This indicates that a critical layer existed for the stability of diblock copolymer thin film under annealing. If the film thickness is thinner than this critical value, no dewetting in the film occurs and the entire silicon is only covered by a polymer brush, which can be seen in the films of 2.3 and 5.8 nm thicknesses. While the film of initial thickness is thicker than the critical thickness, which is equal to half of an interlamellar layer [17, 22], a dense polymer brush adjacent to the Si substrate formed. The excess material cannot interpenetrate into this dense layer but dewets this layer to form droplets.

The interlamellar spacing (*D*), which is a balance between the elastic stretching energy and the interfacial energy contributions [2, 6], is in accordance with

(1) $$\begin{array}{c} D\alpha \chi^{1/6}N^{2/3}, \end{array}$$

where *χ* is Flory-Huggins segmental interaction parameter and *N* is the total degree of polymerization. 

The interlamellar spacing of PEO-*b*-PS in our study can be estimated on the basis of information about a higher molecular weight diblock copolymer with ordered lamellar phase structure. Samples of PEO-*b*-PS with *N* = 285 exhibit  *D* = 18.7 nm [13]. Therefore, for the copolymer with *N* = 154, half of the interlamellar spacing,  *D*/2, is approximated to be about 6.2 nm. This value is in well accordance with the experimental results between 5.8 nm and 10.7 nm, which confirm a reasonable brush thickness.

For the film thickness higher than half of the interlamellar spacing, concerning the composition of PEO-*b*-PS that the volume fraction of PEO blocks (*f*_PEO_) is 0.528 in the melt at 393 K [13], the ordered multilayered lamellae structure parallel to the silicon substrate should be formed in these nearly symmetric compositions if the phase separation occurs between the PEO block and PS block [6]. However, when analyze the cross-section shape of the dropletd by AFM, no terrace lamellae structure with an integral number of interlamellar spacing was observed in the film thicker than 6.2 nm.

To get further insight into this, the thermodynamic compatibility between the PS blocks and PEO block was analyzed. The miscibility between the PEO block and PS block is governed by microphase separation strength (*χN*) for the PEO-*b*-PS diblock copolymer. For the copolymer in the segregation regime (*χN* = 10.5) predicted by mean-field theory, the  *χ*  is about 0.0682. The order-disorder transition temperature (*T*_ODT_) can be evaluated from [13]

(2) $$\begin{array}{c} T=\frac{21.3}{\chi +7.05\times 10^{-3}}\left(T\;\text{in}\;\text{K}\right). \end{array}$$

The calculated value of *T*_ODT_ is 283 K. We note that the glass transition temperature of the PS blocks (*T*_*g*,PS_) is 351 K [35] and the melting temperature of PEO blocks (*T*_*m*,PEO_) is 330 K. Because *T*_*g*,PS_ > *T*_*m*,PEO_ > *T*_ODT_, the diblock copolymer will be vitrified and crystallized above the *T*_ODT_ before phase separation when cooled from the higher temperatures. Since the strong phase separation between PEO and PS blocks only takes place at the temperature below *T*_ODT_ [13], the ordered lamellar structure cannot be achieved in the bulk state of this low molecular weight diblock copolymer (5000–4300). Also, the microphase separation strength *χN* at 393 K is only 7.3. Thus, at annealing temperature of 393 K (above the *T*_*g*,PS_), the phase separation did not occur and this low-molecular-weight diblock copolymer in the droplet is in the disordered state. Therefore, when the PEO block crystallized within the droplets, spherulite is formed instead of the concentric circular superimposed or terraced lamellar layers.

The equilibrium contact angle (*θ*_*e*_) of the PEO-*b*-PS melted on the brush layer can be expressed by Young's equation

(3) $$\begin{array}{c} \gamma_{\text{SV}}=\gamma_{\text{SL}}+\gamma_{\text{LV}}\cos\theta_{e}, \end{array}$$

where *γ*_SV_ is the interfacial energy between the solid and the vapor phase, *γ*_SL_ is liquid/solid interfacial tension, and *γ*_LV_ is the liquid/vapor interfacial tension. From the line scan of height image of a typical droplet in Figure 3(b), the  *θ*_*e*_  is calculated to be approximately 4.0°. This indicates an autophobic dewetting, which corresponds to the inability of a fluid to wet a thin absorbed polymer brush of the same composition [17, 18, 36]. The origin of autophobic behavior of PS-*b*-PEO copolymers is related to the selective interactions between PEO blocks and Si substrate leads to entropy difference between the chain conformations of the adsorbed and free molecules [23, 25]. Thus, the excess destabilized diblock copolymer melt dewet the interlamellar layer adjacent to the Si substrate to form droplet.

**Figure 6:**
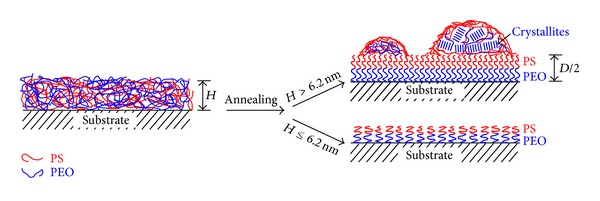
Schematic side view of the PEO-*b*-PS diblock copolymer with different initial thickness developed on the Si wafer under thermal annealing. A polymer brush was formed in the film of thickness less than 6.2 nm. When the film is thicker than 6.2 nm, a dense polymer brush equal to half of an interlamellar layer (*D*/2) was formed, and the excess material dewets this layer to form droplets. The PEO block can crystallize to form spherulite in the bigger droplets.

Figure 6 illustrates the morphology evolution of PEO-*b*-PS film with various thicknesses under thermal annealing. When the initial thickness of the PEO-*b*-PS diblock copolymer film is less than  *D*/2  (half of the interlamellar spacing, 6.2 nm), an ordered brush remains on the substrate with the PEO block adsorbed preferentially onto Si substrate because of the substrate-induced effect. If the initial thickness is higher than *D*/2, a densely packed brush layer with highly stretched chains was formed. Autophobic dewetting occurs, and typical droplets with PS-rich surface are formed. The PEO block inside the bigger droplet can be crystallized to form spherulites.

## 5. Conclusions

We studied the structural evolution of low-molecular-weight PS-*b*-PEO diblock copolymer thin film with various initial film thicknesses on SiOx substrate. After the smooth copolymer films were fully annealed, films of initial thickness less than half of the interlamellar spacing (6.2 nm) form a brush with PEO blocks anchored on the Si substrate due to the strong affinity of PEO block to the SiOx substrate. When the initial film thickness is higher than 6.2 nm, a densely packed brush with highly stretched chains which are equal to half of an interlamellar layer formed on the substrate. The excess material dewet this underlying brush layer to form droplets. The surface PS block was rich in the surface and the PEO block inside the bigger droplet form spherulite.
